# The Role of Sphingolipids on Innate Immunity to Intestinal *Salmonella* Infection

**DOI:** 10.3390/ijms18081720

**Published:** 2017-08-07

**Authors:** Fu-Chen Huang

**Affiliations:** Department of Pediatrics, Kaohsiung Chang Gung Memorial Hospital and Chang Gung University College of Medicine, Kaohsiung 833, Taiwan; huang817@cgmh.org.tw; Tel.: +886-7731-7123 (ext. 8724); Fax: +886-7733-8009

**Keywords:** sphingolipids, *Salmonella*, intestine epithelia, innate immunity

## Abstract

*Salmonella* spp. remains a major public health problem for the whole world. To reduce the use of antimicrobial agents and drug-resistant *Salmonella*, a better strategy is to explore alternative therapy rather than to discover another antibiotic. Sphingolipid- and cholesterol-enriched lipid microdomains attract signaling proteins and orchestrate them toward cell signaling and membrane trafficking pathways. Recent studies have highlighted the crucial role of sphingolipids in the innate immunity against infecting pathogens. It is therefore mandatory to exploit the role of the membrane sphingolipids in the innate immunity of intestinal epithelia infected by this pathogen. In the present review, we focus on the role of sphingolipids in the innate immunity of intestinal epithelia against *Salmonella* infection, including adhesion, autophagy, bactericidal effect, barrier function, membrane trafficking, cytokine and antimicrobial peptide expression. The intervention of sphingolipid-enhanced foods to make our life healthy or pharmacological agents regulating sphingolipids is provided at the end.

## 1. Introduction

*Salmonella* species remain a major public health problem for the whole world. Therefore, it is mandatory to explore alternative therapy to antibiotic treatment in order to reduce both the use of antimicrobial agents and the emergence of drug-resistant *Salmonella*.

In eukaryotic cells, the plasma membrane is in a constant state of equilibrium with the internal cellular membranes through the processes of endocytosis (membrane uptake) and exocytosis (recycling). Proteins and lipids of the plasma membrane assemble into small dynamic subdomains that can be stabilized to form larger specialized microdomains [1]. These sphingolipid- and cholesterol-enriched lipid microdomains (also called lipid rafts) [2] attract signaling proteins and orchestrate them toward cell signaling and membrane trafficking pathways [3,4,5,6]. These reviews have highlighted the important role of such microdomains for the regulation of many biological and pathological processes. Sphingolipids in intestinal mucosal cells may inhibit cholesterol absorption [7]. The paradoxical effects of sphingolipids and cholesterol were demonstrated in our previous studies [8,9,10]. Therefore, the interaction between sphingolipids and cholesterol deserves to be investigated in the future.

Besides their roles in the regulation of many biologic processes and immune responses [11,12], sphingolipids also play a crucial role in infectious diseases [11,13,14]. Beyond the mucosal barrier, intestinal epithelial cells (IECs) mediate innate immunity against pathogenic bacteria, including *Salmonella*. It is therefore mandatory to exploit how membrane sphingolipids act in the innate immunity of IECs to *Salmonella* infection.

The basic structure, metabolic pathway, and bioactive signaling of sphingolipids have been comprehensively reviewed [15,16,17]. Ceramide, sphingomyelin, sphingosine, sphingosine-1-phosphate (S1P), and ceramide-1-phosphate (C1P) have emerged as chief bioactive mediators in the context of sphingolipid biology. Ceramide is in the central part of the sphingolipid metabolic pathway and serves as a critical point in the pathway. It acts as a substrate for complex sphingolipids through the involvement of several enzymes; these include neutral, alkaline, and acidic sphingomyelinases converting sphingomyelin to ceramide; sphingomyelin synthase converting ceramide to sphingomyelin; ceramidase converting ceramide to sphingosine; and glucosylceramide synthase converting ceramide to glycosphingolipids. The metabolism and clinical implications of sphingolipids in the gut have also been reviewed elsewhere [17,18,19]. In this present review, we focus on the role of sphingolipids in the innate immunity of intestinal epithelia against *Salmonella* infection.

## 2. Adhesion

Many pathogens and microbial toxins bind to the glycosphingolipids of the host, most of which are derived from glucosylceramide (GlcCer). A detailed list of pathogens that bind to glycosphingolipids is given in the review by Schengrund [20]. The binding of many pathogens to human cells can be inhibited by the following methods: (1) using substances coating the host’s glycosphingolipids to compete with the pathogen for binding; (2) using glycosphingolipid-like substances (decoys) to displace pathogens from the binding sites (adhesins); or (3) depleting the host cells of their surface glycosphingolipids [21]. *Clostridium difficile* toxin-induced host cell membrane protrusions were demonstrated to be involved in enhanced bacterial adhesion and colonization. The lipid microdomains are essential for the membrane protrusions [22]. Anti-adherence and bactericidal activities of sphingolipids against *Streptococcus mutans* were also reported [23]. The importance of glycosphingolipids in *Salmonella* infection was discovered in a patient with Gaucher disease, a sphingolipidosis characterized by abnormal accumulation of glucocerebroside in cells of the monocyte–macrophage system due to inadequate GlcCer glucosidase [24].

## 3. Barrier Function

Sphingolipids present in the intestinal mucosa create a nonspecific barrier and in that way protect enterocytes against digestive enzymes, bile salts, or acidic gastric juices. Dysfunction of these mechanisms can result in the development and progression of inflammatory diseases. In a porcine model, inhibition of de novo ceramide synthesis with the mycotoxin fumonisin B1 altered the proliferation and barrier function of IECs, which in turn led to the induction of inflammation [25]. Bock et al. [26] proved that exogenous sphingomyelinase causes deterioration of the intestinal barrier function and increases inflammation as a result of the reduction of sphingomyelin in mucosal cells.

On the other hand, sphingolipids play important roles in membrane structure and cell function. Sphingomyelin is required for membranous translocation of Ras homolog family member A (RhoA) and cell division cycle 42 (Cdc42), resulting in caveolar endocytosis [27]. The inhibitory effects of general sphingolipid depletion on endocytosis could be partially reversed by incubating cells with exogenous sphingomyelin. However, activation of geranylgeranylated proteins, including Ras-related C3 botulinum toxin substrate-1 (Rac1) and Cdc42, is critical for disruption of the barrier integrity by *S. typhimurium* [28]. It is reasonable to investigate the pros and cons of sphingolipids on the disruption of epithelial barrier integrity by *Salmonella* infection.

## 4. Membrane Trafficking

Although most prokaryotic cells do not contain sphingolipids, some bacteria have evolved mechanisms by which they can utilize the host sphingolipids to promote their pathogenicity. Intracellular bacterial pathogens have developed many strategies to ensure their own survival and to evade the host immune system by hijacking ceramide-enriched lipid rafts of the host [29]. Other microbes are able to utilize host sphingolipids for incorporation into inclusions in which replication occurs, as in the case of *Chlamydia trachomatis*.

Despite the fact that sphingolipids may aid microbial pathogenesis, they may play key roles in the host’s defenses against infections. For extracellular pathogens, sphingolipid-enriched rafts facilitate the phagocytosis and eventual lysis of *Pseudomonas aeruginosa* [14]. In the case of intracellular pathogens, the host may initiate sphingolipid-mediated pathways that enable containment of the microbe or enhance their clearance, such as *S. typhimurium*. *S. typhimurium* pathogenicity island 2 (SPI-2) type III secretion system (T3SS) allows delivery of bacterial effector proteins into the cytoplasm of the host cells to trigger actin polymerization and mediate epithelial cell invasion via Rho GTPases Rac1 and Cdc42 [30]. Once inside the host cells, *S. typhimurium* is enclosed in *Salmonella*-containing vacuole (SCV). Disruption of the SCV then leads to increased replication of *S*. *typhimurium* in the cytosol of the epithelial cells [31]. One of the *Salmonella* effector proteins, cell invasion protein (SigD)/plasma partitioning protein (SopB), is essential for establishment of the SCVs [32], as well as protein kinase B (Akt) activation by *Salmonella* in HeLa cells [33], which promotes intracellular survival of the bacteria. *Salmonella*-induced cholesterol accumulation in SCVs activates the PI3K/Akt pathway. This process may subsequently protect IECs from apoptosis, which may contribute to the proliferation of *Salmonella* in IECs [8]. The vesicle COPI complex may affect the membrane sorting of cholesterol and ganglioside GM1, bind activated Rac1 and Cdc42, resulting in membrane ruffling and *Salmonella* invasion [34].

Mononuclear phagocytes constitute a critical component in the resistance to *Salmonella*. The NADPH phagocyte oxidase and inducible nitric oxide synthase in activated peritoneal macrophages [35,36] exert microbial killing of the internalized *Salmonella* cells in the early and late stages of salmonellosis. Constitutive acid sphingomyelinase optimizes early macrophage killing of *Salmonella* by acting synergistically with a functional NADPH oxidase [37]. Besides this, *Salmonella* triggers a significant increase in the secreted fraction of acid sphingomyelinase, which may be required for the bactericidal activity of murine macrophages against a variety of pathogenic microorganisms.

Myosin motor proteins are known to regulate the dynamic organization of the plasma membrane by generating and maintaining an actin-dependent force on the cell surface and by transporting and fusing membrane vesicles with it. Myosin-1c (MYO1C) is expressed in the plasma membrane of most eukaryotic cells. MYO1C promotes lipid-raft-enriched membrane tubule formation and facilitates recycling of lipid raft membrane and proteins to control cell spreading, migration, and *Salmonella* invasion [38].

## 5. Bactericidal Effect

The bactericidal activities of milk lipids, including sphingosine and ceramide, have been confirmed on the pathogenic bacterial strains *Campylobacter jejuni*, *Escherichia coli* O157:H7, *Listeria monocytogenes*, and *Salmonella enteritidis* in vitro [39]. The antibacterial activity of sphingosine is not limited to *Staphylococcus aureus* and has been demonstrated against various Gram-positive and Gram-negative bacteria [40,41], including *P. aeruginosa*, *Haemophilus influenzae*, *Acinetobacter baumannii*, *Moraxella catarrhalis*, and *Burkholderia cepacia* [42,43].

## 6. Autophagy

Two reports have disclosed clinical implications of sphingolipids in the bacterial infections [11,14]. Increasing evidence illustrates the critical role of autophagy in controlling infections by directing intracellular or ingested pathogens to phagosomes, resulting in microbial elimination [44]. Sphingolipids are a class of bioactive lipids that mediate many key cellular processes, including apoptosis and autophagy [6,45]. They might act as potential targets for therapeutic intervention in human diseases [46]. For instance, sphingolipid synthesis is involved in autophagy in *Saccharomyces cerevisiae* [47]. De novo biosynthesis of sphingolipids has been shown to be essential for the induction of autophagy, because autophagosome formation was eliminated by myriocin (an inhibitor of the critical enzyme serine palmitoyltransferase in de novo sphingolipid synthesis) in Toll-like receptor-4 (TLR4) agonist-stimulated RAW264.7 macrophages [48].

Focal adhesion kinase activated by *Salmonella* is recruited to SCVs, where it promotes robust Akt activation and stimulation of the mammalian target of rapamycin (mTOR) signaling pathway, leading to suppression of autophagy and enhanced bacterial survival in macrophages [49].

Recent evidence demonstrates that *Salmonella* can actively suppress autophagy to promote its intracellular survival in epithelial cells [50]. In the early phase of infection, *Salmonella* triggers acute intracellular amino acid starvation and mTOR inhibition, resulting in the induction of autophagy. However, in the later phase of infection, the rapid normalization of cytosolic amino acid levels in *Salmonella*-infected cells reactivates mTOR at the surface of the SCV and favors bacterial escape from autophagy. On the other hand, ubiquitination serves as a signal to trigger nuclear factor-κB (NF-κB) activation, which then dampens bacterial proliferation by inducing inflammation. Ubiquitination also marks bacteria that escape to the cytosol for autophagosome-mediated degradation by tagging them with a dense polyubiquitin coat [51]. The host ubiquitinome [52,53] dataset might serve as a resource to reveal targets for the inhibition of *Salmonella* invasion and inflammation. The sphingolipid ceramide modulates the human ether-a-go-go-related gene (HERG) potassium channel in the cell membrane of HEK293 cells by targeting ubiquitylated proteins in the cytosol for lysosomal degradation [54]. Neutral sphingomyelinase-2 regulates interleukin-1 receptor-associated kinase-1 (IRAK-1) protein ubiquitination and degradation [55] in response to interleukin-1β. The effects of sphingolipids on the ubiquitination of *Salmonella* deserve to be investigated in the future.

Inhibition of Akt signaling [56,57] and enhancement of extracellular signal-regulated kinase (ERK) 1/2 activity [56,58] were associated with macroautophagy in human colon adenocarcinoma cells, such as HT29 and HCT-15. Activated ERK upregulated Beclin-1 expression through induction of B-cell lymphoma-2 (Bcl-2) phosphorylation and thereby induced autophagy [59]. Inhibition of cellular sphingolipids by myriocin abrogated autophagy via activation of Akt and downregulation of Beclin-1 [60]. Activation of the c-Jun NH2-terminal kinase (JNK) pathway was involved in ceramide-induced autophagy and the regulation of Beclin-1 [61] and autophagy protein LC3 expression [62]. Beclin-1 and LC3-II are known biological markers of autophagy. On the other hand, the involvement of sphingolipid synthesis on autophagy in *Saccharomyces cerevisiae* [47] and in mammalian cells [60] was illustrated previously. The interaction of NOD2 and ATG16L1 in epithelial cells activates autophagic clearance of *Salmonella* [63]. It is reasonable to investigate whether membrane sphingolipids play a role in *Salmonella*-induced autophagy in IECs and the involvement of these signaling pathways. We observed that inhibition of de novo sphingolipid synthesis with myriocin enhanced Akt but suppressed ERK phosphorylation, and repressed the membrane recruitment of NOD2 and ATG16L1, leading to decreased *Salmonella*-induced LC3-II autophagy expression [10]. It suggests that membrane sphingolipids may be involved in the *Salmonella*-induced cellular autophagy of damaged SCVs, because the suppressed Akt phosphorylation may contribute to the disruption of the SCVs, which is directed for autophagic clearance. Plasma membrane cholesterol mediates PI3K/Akt-dependent anti-inflammatory and anti-apoptotic responses, resulting in SCV formation [8,64], suggesting that sphingolipids may play a role contrary to that of membrane cholesterol on SCVs.

A previous report [65] that fumonisin B1, a ceramide synthase inhibitor, increases intestinal colonization by pathogenic *E. coli* in pigs supports our observation. The abnormalities in the handling of intracellular bacteria by autophagy might also play a role in Crohn’s disease pathogenesis [63,66,67]. The impaired autophagy of intracellular *Salmonella* by the Crohn’s disease-associated ATG16L1 variant implicated in disease pathogenesis [67]. The involvement of sphingolipid in inflammatory bowel disease (IBD) may include mucosal integrity, barrier, receptor functions and formation of sphingolipid messengers in epithelial and inflammatory cells. Sphingolipid-enhanced foods or pharmacological agents may induce autophagic clearance of invading pathogens and lower the risk of Crohn’s disease.

## 7. Interleukin-8

IECs act not only as a barrier to bacterial colonization in the gut, but also as an integral component of the mucosal innate immunity of the host through its secretion of inflammatory chemokines (e.g., interleukin-8 (IL-8)), and antimicrobial peptides (e.g., human β-defensins (hBDs)) to defend against *Salmonella* invasion. IL-8 released from the infected epithelial cells recruits neutrophils to contain and eliminate the invading pathogen. However, the accumulation of neutrophils brings on characteristic pathological changes of colitis [68]. In contrast, antimicrobial peptides (hBDs) will defend against and kill *Salmonella*. A comprehensive review on sphingolipids in inflammation has been given by Nixon [69]. In this section, we focus instead on the role of sphingolipids in IL-8 and hBD-2 induction.

The activity of NF-κB, a central regulator of proinflammatory responses, was increased by ceramide in the IECs. Ceramide may be required for lipid raft TLR complex formation in response to bacterial toxins, such as lipopolysaccharide (LPS) [70]. Ceramide was identified as a TLR4 agonist. Microbial ligands with glycosphingolipid specificity (P fimbriae or the B subunit of Shiga toxin) were shown to increase the levels of ceramide and to trigger a TLR4-dependent response in epithelial cells [71]. S1P secreted from airway epithelial cells indirectly promoted neutrophil recruitment by increasing IL-8 production and intercellular adhesion molecule-1 (ICAM-1) expression in A549 alveolar epithelial cells [72]. However, ceramide formed by neutral sphingomyelinase was reported to inhibit the LPS-induced IL-8 response in aortic endothelial cells in response to phospholipid oxidation products [73].

Ceramide plays an important role in several signaling pathways by triggering a cluster of receptor molecules in membrane rafts [74]. Increased ceramide levels give rise to the formation of ceramide-enriched membrane rafts and the activation of specific signaling pathways, and the release of cytokines from infected mammalian cells [11]. In response to *Helicobacter pylori* infection, ceramide and TLR4 are mobilized into membrane rafts in gastric epithelial cells, whereby they activate TLR4 signaling and contribute to NF-κB activation and IL-8 production [75]. Diminished ceramide production by inhibitors augmented tumor necrosis factor-alpha (TNF-α)-induced IL-8 production [76] from A549 respiratory epithelial cells. In the respiratory epithelium, TNF-α induces ceramide accumulation. Ceramide activates PP2A, which deactivates the JNK, p38, and ERK/mitogen-activated protein kinase (MAPK) pathways, resulting in the reduction of ongoing IL-8 production [76]. Acid sphingomyelinase, a key enzyme in ceramide-generating sphingolipid metabolism, can be activated by various cellular stressors, including bacterial pathogens. The defective acid sphingomyelinase pathway is associated with an overwhelming IL-8 response, decreased bacterial uptake, and reduced apoptotic response in cystic fibrosis with *P. aeruginosa* infection [77]. In contrast, LPS efficiently increases the release of IL-8 from HT-29 IECs by activating neutral sphingomyelinase, with subsequent hydrolysis of sphingomyelin to ceramide and activation of NF-κB in the cells, resulting in colitis in mice [78].

Induction of ceramide by LPS in alveolar macrophages results in activation of the PI3K/Akt pathway, preventing ceramide-induced apoptosis. On the other hand, ceramide-activated protein phosphatases, including PP2A, have been shown to inhibit Akt by dephosphorylation of serine residues [79,80] and to modulate key cellular processes, including exocytosis, alternative pre-mRNA splicing, and glycogen metabolism. Raft nanodomains trigger the PI3K/Akt signaling pathway, by facilitating Akt recruitment and activation in the plasma membrane [81].

Because ceramide can activate the class of molecules that inhibit the PI3K/Akt signaling pathway, which negatively regulates *Salmonella*-induced IL-8 production [17], it was supposed to suppress *Salmonella*-induced IL-8 production in IECs. It would be worthwhile to study the suppressive role of sphingolipids on *Salmonella*-induced IL-8 production in IECs and subsequently on colitis. We observed that inhibition of de novo sphingolipid synthesis with myriocin suppressed ERK but enhanced Akt activation [8], resulting in the suppression of *Salmonella*-induced IL-8 production (unpublished). It suggests that the de novo synthesis of sphingolipids may enhance IL-8 production in *Salmonella*-infected IECs, which is also in contrast to the response seen with membrane cholesterol [8,64].

## 8. Human β-Defensin

In atopic dermatitis, the defense system of the skin against bacterial invasion is significantly disrupted. The first defender of the epidermal innate immune response is the antimicrobial peptides, which exhibit broad-spectrum antimicrobial activity against multiple pathogens. Increased S1P levels strongly stimulated cathelicidin antimicrobial peptide expression, which elevated the antimicrobial activity against multiple pathogens, resulting in improvement of the atopic dermatitis in afflicted patients [82]. The endoplasmic reticulum stress-initiated C1P regulates epithelial innate immunity by stimulating BD production, including hBD-2 and hBD-3 [83]. Exogenous C1P also increases hBD-2 and hBD-3 production. Conditioned medium from C1P-stimulated keratinocytes showed antimicrobial activity against *Staphylococcus aureus*. Following infection with *Candida*, gingival epithelial cells expressed high levels of antimicrobial peptides, including hBD-1, hBD-2, and hBD-3, to achieve their defense against the infecting agent. Disruption of the sphingolipid biosynthetic gene *IPT1* affected *Candida*–host interaction, thus preventing TLR activation and BD expression [84].

NOD2 serves as an intracellular pattern recognition receptor to mediate the induction of the antimicrobial peptide hBD-2 [85]. HBD-2 exhibits a broad spectrum of antimicrobial activity to kill bacteria in vivo [86] and is induced in various epithelia (e.g., skin, respiratory tract, digestive tract, and genitourinary tract) upon extracellular as well as intracellular bacterial challenge, suggesting that it is important in the host defense against microbes in the gut. Lipid rafts attract signaling proteins and move them to locations for subsequent signaling through intracellular trafficking [3]. Membrane targeting of NOD2 in IECs is required for NOD2-dependent NF-κB signaling [87] and subsequent production of antimicrobial peptide hBD-2. Accordingly, we demonstrated that *Salmonella* induces NOD2 recruitment into the membrane, whereas myriocin suppresses this membrane recruitment of NOD2 and subsequently hBD-2 expression as well [10]. As mentioned in the autophagy section, the interaction of NOD2 and ATG16L1 in epithelial cells activates autophagic clearance of *Salmonella.* It suggests that sphingolipids-mediated recruitment of NOD2 and Atg16L1 into the plasma membrane of *Salmonella*-infected IECs contributes to the extracellular killing and intracellular autophagic clearance of the invading pathogen.

An increased risk of IBD following enteric infections with *Salmonella* was observed [88]. Dysregulation of the NOD2-mediated defense against enteric bacteria (e.g., pathogenic adherent-invasive *E. coli*) in patients with NOD2 mutations and dysfunction of mucosal hBD-2 may play an important role in the pathogenesis of IBD [66,89,90]. Children with Crohn’s disease also showed a lower expression of hBD-2 in the inflamed terminal ileum and ascending colon [91]. FTY720, a synthetic sphingosine analog of myriocin, caused a specific downregulation of proinflammatory signals, while simultaneously inducing functional activity of CD4^+^CD25^+^ Treg [92]. It suggests that FTY720 offers a promising new therapeutic strategy for the treatment of IBD. Thus, the role of membrane sphingolipids in IBD merits investigation in vivo.

## 9. Sphingolipid-Enhanced Foods

The level of sphingolipids is over two-fold higher in IECs of the small intestine than in the colonic mucosa [18]. The estimated daily requirement of sphingolipids for gastrointestinal mucosal recovery is about 1.5 g [93]. In the intestinal villi, sphingolipids are located mainly in the apical membrane and to a minor extent in the basolateral membrane [94]. Sphingolipids are either delivered to the mucosal cells through diet or synthesized via the de novo pathway [18]. In the gastrointestinal tract, sphingolipids are synthesized mainly in the de novo pathway, where the first reaction is catalyzed by serine palmitoyltransferase. The total amounts of sphingolipids in foods vary considerably, from a few micromoles to several millimoles per kilogram. Foods that are especially rich in sphingolipids include dairy products, particularly milk and eggs. Milk fat consumption was shown to be related to a reduced number of foodborne infections [39]. Gangliosides are the most significant sphingolipids since they contribute to proper central nervous system growth and protect from infections in the gut during infancy [27] through the binding and inactivation of bacterial toxins [95,96]. Gangliosides act as co-receptors with TLR5 for flagellin (FliC) and promote hBD-2 expression via mitogen-activated protein kinase. Moreover, gangliosides present in human milk can stimulate the growth of probiotic bacteria, such as *Bifidobacterium* [97]. Dietary ganglioside reduces proinflammatory signaling in the intestine [98] and its content in the intestinal mucosa can be increased through dietary intake.

## 10. Conclusions

**Figure 1:**
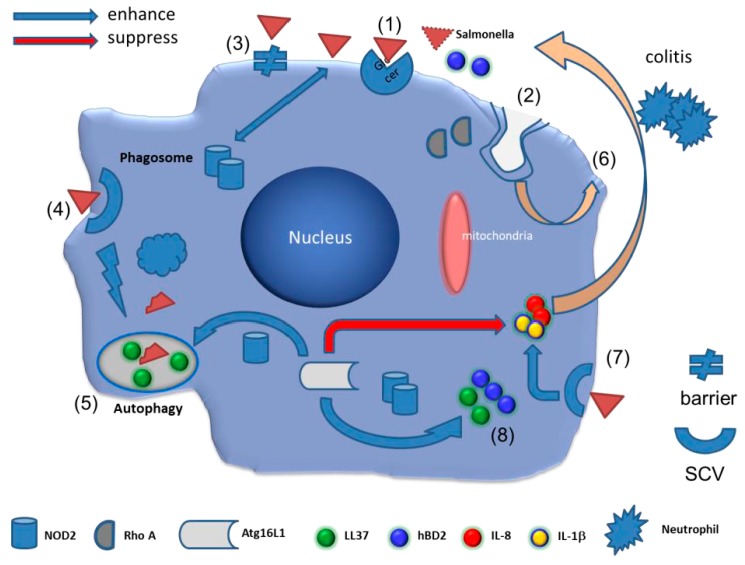
The role of sphingolipids on innate immunity of intestinal epithelial cells against *Salmonella* infection. After *Salmonella* infection, intestinal epithelial cells mediate innate immunity against pathogenic bacteria. The reported mechanisms were summarised in this cartoon module. (**1**) *Salmonella* binds to the glycosphingolipids (e.g., glucosylceramide) of the host, leading to adhesion of the bacteria; (**2**) Sphingomyelin is required for membranous translocation of RhoA and Cdc42, resulting in caveolar endocytosis. (**3**) However, activation of Rac1 and Cdc42 is critical for the disruption of the barrier integrity by *S.* *typhimurium* via altered localization of tight and adherens junction proteins; (**4**) One of the Salmonella effector proteins, SigD/SopB, is essential for the establishment of the *Salmonella*-containing vacuoles (SCVs) which promotes intracellular survival of the bacteria. Disruption of the SCV then leads to increased replication of *S*. *typhimurium* in the cytosol of the epithelial cells; (**5**) Membrane sphingolipids may be involved in the *Salmonella*-induced cellular autophagy of damaged SCVs, because the suppression of phosphorylated Akt may contribute to apoptosis of the SCVs, leading to their damage, which is directed for autophagic clearance; (**6**) MYO1C regulates lipid raft recycling to the cell surface to deliver signaling components and the extra membrane essential for cell surface expansion and remodeling for controlling cell spreading, migration, and *Salmonella* invasion; (**7**) The de novo synthesis of sphingolipids may enhance IL-8 production in *Salmonella*-infected IECs; (**8**) In contrast, sphingolipids recruiting NOD2 and Atg16L1 into the plasma membrane of IECs infected by *Salmonella* contribute to the enhanced autophagic clearance and hBD-2-killing of the invading pathogen.

Studies showing that feeding experimental animals with sphingolipids inhibits colon carcinogenesis and atherosclerosis [17], suggest that sphingolipids represent a “functional” constituent of food. However, the functional importance of sphingolipids on *Salmonella* infection has rarely been investigated. To battle with *Salmonella* infection, the homeostasis between cholesterol and sphingolipids may affect the innate immunity of the host to defend against the invasive bacteria [8,9,10,99]. These novel and promising findings provide a therapeutic strategy to enhance sphingolipids over cholesterol in order to enrich the innate immunity against *Salmonella* infection. Because the manipulation of sphingolipids in host cells can affect pathogen infection and inflammation (summarized in Figure 1), sphingolipids-enhancing pharmacological agents or sphingolipids-enriched diets [17] could become alternative treatment of infectious or inflammatory bowel diseases in the future.
